# Severe SARS-CoV-2 Infection and Campylobacter coli Bacteremia in a Patient With Good’s Syndrome

**DOI:** 10.7759/cureus.98264

**Published:** 2025-12-01

**Authors:** Magda Gonçalves, Rute Aleixo, José Pinho, Gonçalo Cruz, Cristina Valente

**Affiliations:** 1 Infectious Diseases, Unidade Local de Saúde de Coimbra, Coimbra, PRT; 2 Infectious Diseases, Unidade Local de Saúde de Trás-os-Montes e Alto Douro, Vila Real, PRT

**Keywords:** autoimmunity, campylobacter coli bacteremia, good’s syndrome, hypogammaglobulinemia, immunoglobulin, lichen planus, recurrent infections, sars-cov-2, thymoma

## Abstract

Good’s syndrome (GS) is a primary immunodeficiency characterized by thymoma-associated hypogammaglobulinemia, leading to recurrent infections. A 71-year-old woman, with a history of oral and perineal lichen planus, chronic sinusitis, recurrent otitis, and thymoma excision in 2021, presented with fever and diarrhea after testing positive for SARS-CoV-2. On admission, she appeared stable but had bleeding lichen planus lesions and required supplemental oxygen for moderate respiratory insufficiency. Laboratory results revealed leukocytosis with neutrophil predominance and mild inflammation. A diagnosis of GS was made based on the history of thymoma, detection of hypogammaglobulinemia, and an imbalance in cellular immunity. Additionally, blood cultures grew *Campylobacter coli*, and she completed a 14-day regimen of azithromycin. After completing the antibiotic course, her respiratory condition worsened with progressive bilateral infiltrates seen on X-ray. The polymerase chain reaction-multiplex respiratory pathogen panel was positive only for SARS-CoV-2. Despite mechanical ventilation, treatment with broad-spectrum antibiotic therapy, and immunoglobulin replacement, her condition continued to deteriorate. The patient’s clinical deterioration was attributed to the combined immunodeficiency from GS and the co-infection of SARS-CoV-2 and *C. coli* bacteremia. At this point, SARS-CoV-2 remained detectable through the multiplex respiratory panel, and inflammatory markers were elevated, with a C-reactive protein of 21.13 mg/dL, procalcitonin of 0.69 ng/mL, and leukocytosis of 11.7 × 10⁹/L with neutrophilia (10.51 × 10⁹/L). This case emphasizes the risk of severe infections in patients with GS, as well as the importance of early detection and comprehensive management of concurrent bacterial and viral infections in immunocompromised individuals.

## Introduction

Good’s syndrome (GS) is a rare primary immunodeficiency characterized by the association of thymoma with hypogammaglobulinemia, often associated with autoimmune diseases, resulting in a predisposition to recurrent infections. This clinical condition results in a profound impairment of the immune response, affecting both humoral and cellular immunity. The humoral deficiency, primarily driven by hypogammaglobulinemia, compromises B-cell function, leading to reduced antibody production and an increased susceptibility to encapsulated bacterial infections. Concurrently, cellular immunity dysfunction involving both T-cell and B-cell dysregulation further impairs immune surveillance and pathogen clearance. The defective T-cell response, often associated with thymoma-related abnormalities, undermines the activation and coordination of adaptive immunity, increasing vulnerability to viral and fungal infections [1]. Additionally, the immunological dysfunction associated with GS leads to failure in the defense mechanisms against opportunistic pathogens. Thymectomy and immunoglobulin replacement are the main therapeutic strategies [2].

The profound immune dysregulation in GS underscores the importance for clinicians to maintain a high index of suspicion for infections, as these patients may develop severe, atypical, or rapidly progressive infectious complications. Early recognition of infection and a detailed diagnostic investigation are crucial for the timely initiation of targeted therapy and improved outcomes.

Infections by *Campylobacter coli *are typically associated with self-limiting gastroenteritis [3], but can become very severe in immunocompromised patients, particularly those undergoing immunosuppressive treatments such as corticosteroids. These patients are at risk of developing complications, including the spread of *C. coli *into the bloodstream, leading to invasive infections [4,5].

The COVID-19 pandemic brought further difficulties for immunocompromised patients, with evidence suggesting that COVID-19 may lead to prolonged viral shedding, severe disease, and secondary infections in this population [6,7]. The combination of COVID-19 and bacterial superinfection represents a significant challenge, particularly in the setting of GS, where immune dysregulation may contribute to worse outcomes [1].

This article presents the case of a 71-year-old patient with GS who developed *C. coli* bacteremia with COVID-19 co-infection. This case highlights the complex interaction between bacterial and viral infections in immunocompromised hosts, emphasizes the need for early suspicion and rigorous diagnostic evaluation, and illustrates the importance of intensive monitoring and personalized therapeutic approaches.

## Case presentation

**Figure 1:**
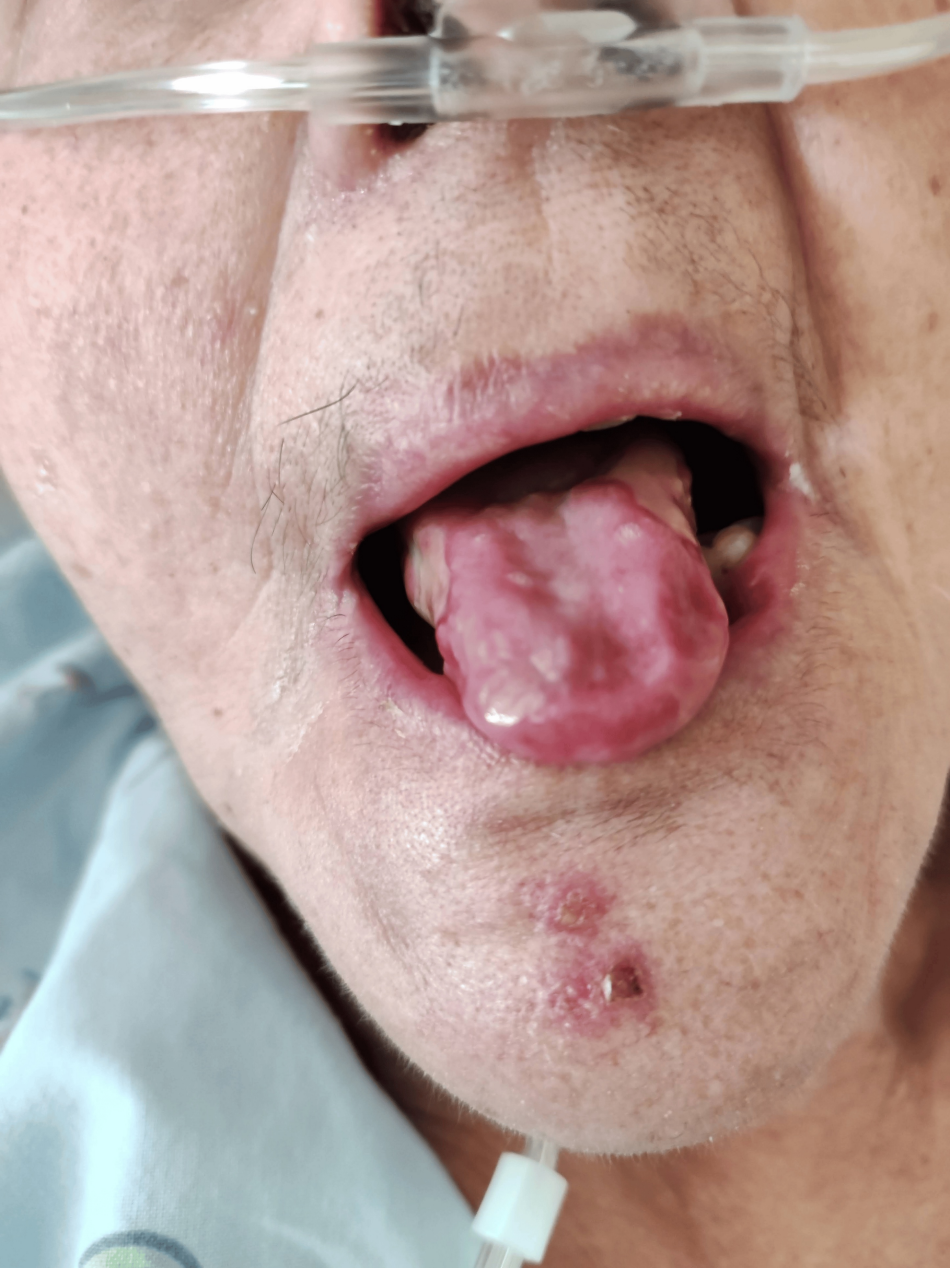
Erosive lichen planus: ulcerated lesion in the lateral area of the tongue with erythematous borders.

A 71-year-old woman with a history of oral and perineal lichen planus (Figure 1), chronic sinusitis, recurrent otitis, and thymoma excision in 2021 was admitted to the emergency department on January 15, 2022. She had been in isolation since December 30, 2021, following a positive SARS-CoV-2 test. The patient had already received a total of three doses of the COVID-19 vaccine before being admitted. Her regular medications included methylprednisolone (24 mg/day), methotrexate (2.5 mg five times a week), and folic acid (5 mg twice a week) for her autoimmune disease.

The diagnosis of GS was made during hospitalization. A history of thymoma excision in 2021 and recurrent infections, along with an autoimmune-associated condition, raised suspicion. The subsequent detection of hypogammaglobulinemia, confirmed by laboratory tests demonstrating decreased levels of all immunoglobulin types, confirmed the diagnosis when associated with the history of prior thymoma (Table 1). Additionally, an imbalance in cellular immunity was observed, particularly in CD4+ and CD8+ T-cell lymphocytes, with decreased absolute counts (CD4+ 101/mm³ and CD8+ 261/mm³) and an inversion of the CD4+/CD8+ ratio (Table 2). Total lymphocyte populations were also reduced, and, together with hypogammaglobulinemia, this suggested decreased B-cell counts; however, specific peripheral B-cell subset analysis was not performed during hospitalization. Serology for human immunodeficiency virus was negative, and other screening tests were unremarkable for other autoimmune diseases.

**Table 1:** Humoral immunity: differential immunoglobulin count.

Immunoglobulins	Value	Reference range	Unit
Immunoglobulin G	<1.08	5.52–16.31	g/L
Immunoglobulin G1	0.60	4.05–10.11	g/L
Immunoglobulin G2	0.21	1.69–7.86	g/L
Immunoglobulin G3	0.04	0.11–0.85	g/L
Immunoglobulin G4	0.01	0.03–2.01	g/L
Immunoglobulin A	0.06	0.69–5.17	g/L
Immunoglobulin M	<0.05	0.33–2.93	g/L
Immunoglobulin E	<16	<100	UI/mL
Kappa light chains	0.43	4.06–14.45	g/L
Lambda light chains	0.23	2.06–7.69	g/L
Relation kappa/lambda	1.86	1.30–2.61	NA

**Table 2:** Cellular immunity: CD4+ and CD8+ T-cell lymphocyte populations.

Lymphocyte populations	Value	Reference range	Unit
Total lymphocytes	484.0	1,000–4,800	cells/mm³
CD3	89.0	60–85	%
CD3+/CD4+	29.8	30–60	%
CD3+/CD8+	58.4	15–40	%
CD4/CD8	0.39	1.0–1.5	NA
CD3 (absolute)	371	1,100–1,700	/mm³
CD3+/CD4+ (absolute)	101	700–1,100	/mm³
CD3+/CD8+ (absolute)	261	500–900	/mm³

**Figure 2:**
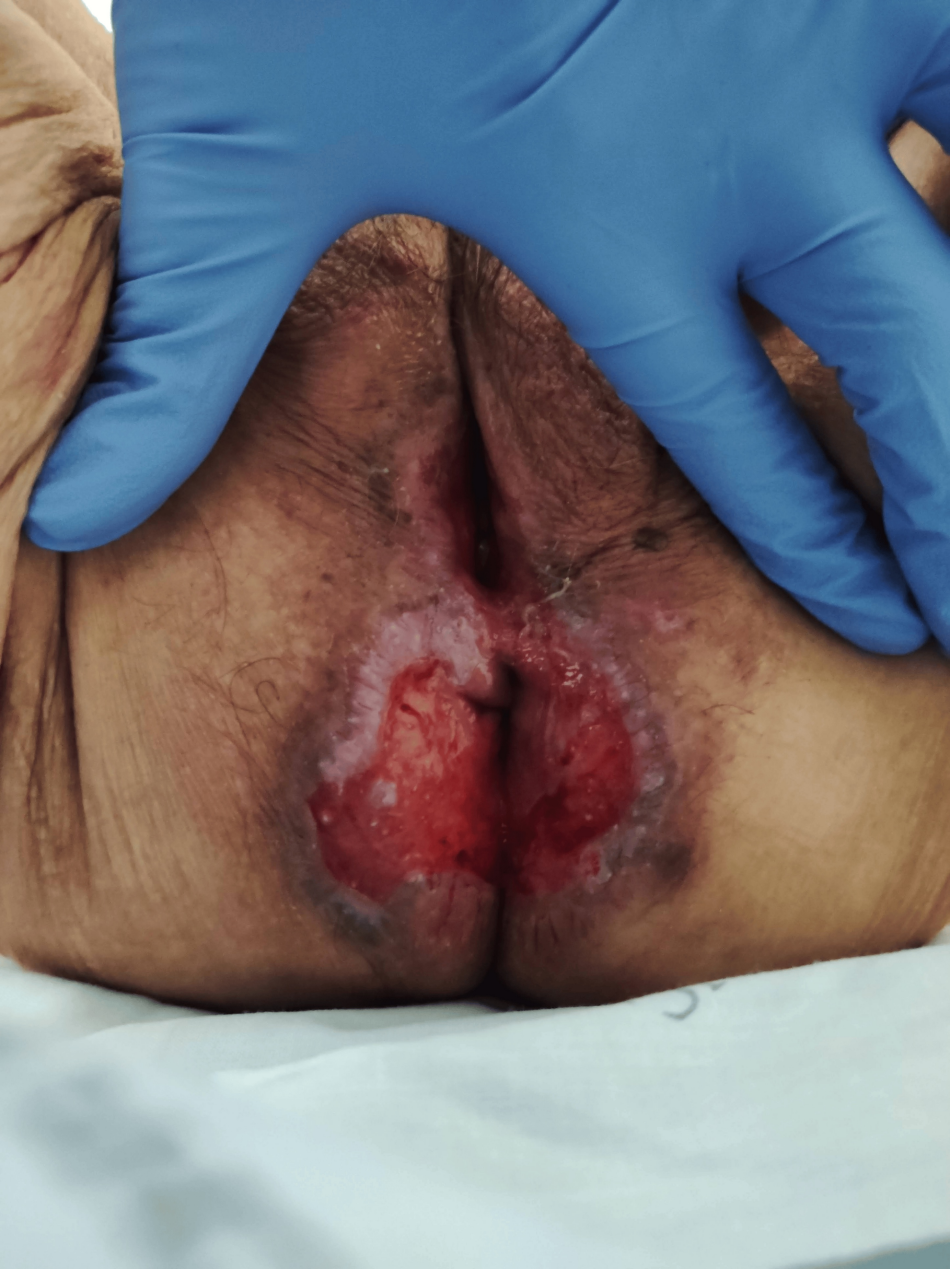
Lichen planus perianal: bleeding lichen planus lesions in the perianal area.

Upon admission, despite being hemodynamically stable, she had fever (maximum temperature of 39°C) and gastrointestinal symptoms such as yellowish, liquid stools and bleeding lichen planus lesions in the perineal area (Figure 2). Laboratory results showed mild elevation of inflammatory markers, with leukocytosis and neutrophil predominance.

**Figure 3:**
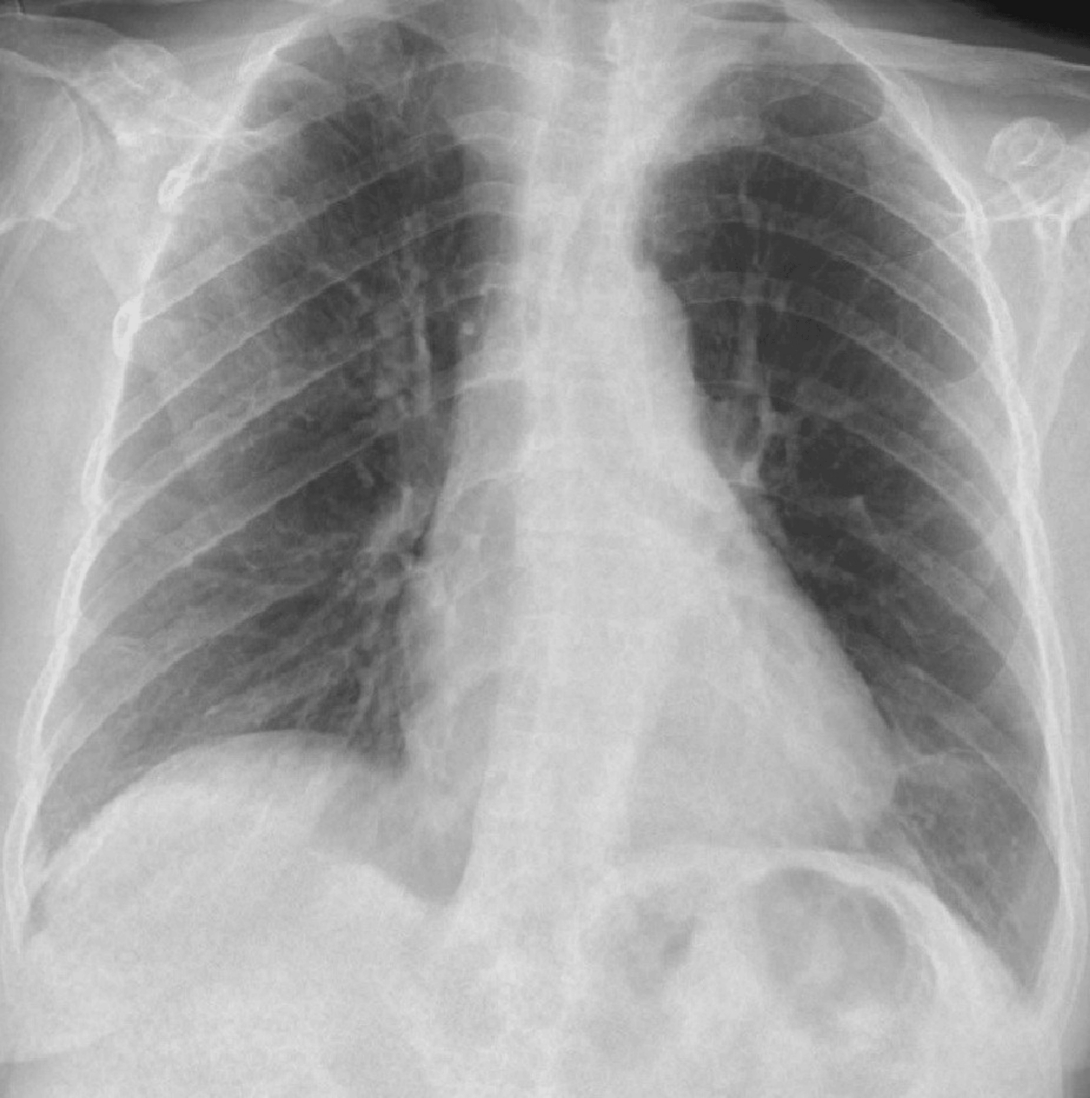
Chest X-ray on the day of admission. Normal chest X-ray showing no significant radiographic abnormalities.

Arterial blood gas analysis revealed a partial pressure of oxygen (PaO₂) of 79.1 mmHg and a partial pressure of carbon dioxide (PaCO₂) of 29.6 mmHg, prompting the initiation of supplemental oxygen therapy and dexamethasone. At this stage, there were no significant radiological findings (Figure 3). Following specimen collection for cultures and molecular diagnostics, the patient was admitted to the COVID-19 ward.

During hospitalization, the patient remained febrile but no longer had diarrhea. Blood cultures collected on the day of admission grew *C. coli*, and inflammatory markers were elevated, with a C-reactive protein of 8.58 mg/dL and leukocytosis of 11.6 × 10⁹/L with neutrophilia (9.93 × 10⁹/L). She was subsequently started on a 14-day course of azithromycin, guided by antibiotic susceptibility testing.

**Figure 4:**
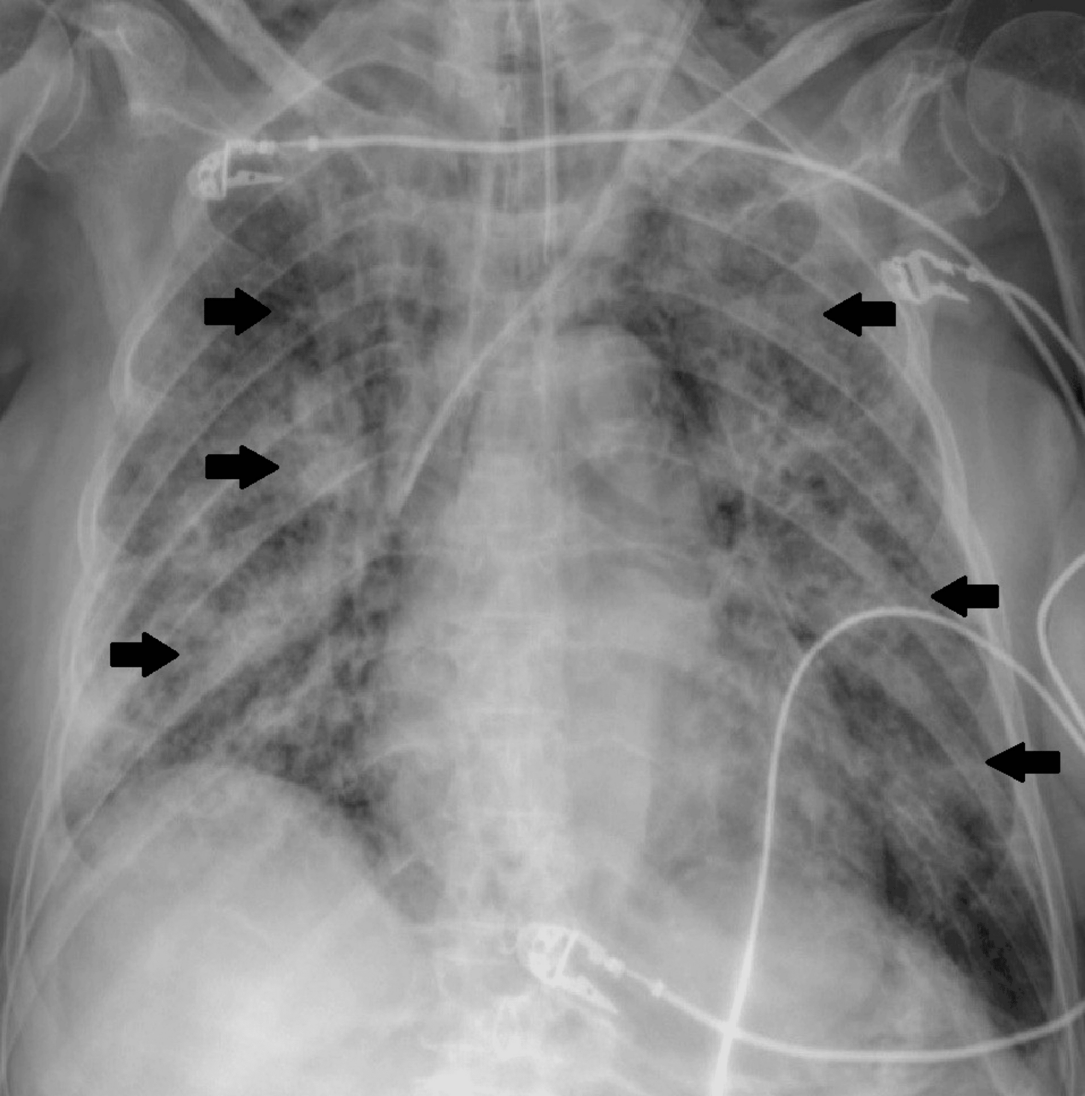
Chest X-ray after worsening of clinical condition. The image shows pneumonia COVID-19 and diffuse lung opacities.

After completing the antibiotic regimen, the patient’s condition worsened, with respiratory deterioration requiring an increase in oxygen therapy. Radiological findings revealed an infiltrate in the right lower lobe, which progressed to bilateral infiltrates on follow-up imaging (Figure 4). Although sputum cultures and repeated blood cultures did not yield any microorganisms, due to suspected bacterial superinfection, she was started on meropenem and linezolid.

On the 20th day of hospitalization, SARS-CoV-2 polymerase chain reaction (PCR) remained positive in bronchoalveolar lavage. At this stage, replacement therapy with intravenous immunoglobulin G (20 g) was initiated, and the patient remained on prophylactic cotrimoxazole. The patient’s condition continued to deteriorate, leading to her transfer to the COVID-19 intensive care unit, where invasive mechanical ventilation was initiated. Diagnostic investigations were performed, including blood cultures and bacteriological analysis of the bronchial aspirate with multiplex PCR panels for respiratory infections, including *Pneumocystis jirovecii*, without identification of any other microorganism, except for persistent SARS-CoV-2 PCR in the bronchial aspirate. Despite all efforts, the patient passed away on the 31st day of hospitalization.

## Discussion

GS is a rare primary immunodeficiency syndrome that typically manifests with hypogammaglobulinemia and recurrent infections, often associated with thymoma [1]. The cornerstone of treatment for GS includes thymectomy and immunoglobulin replacement, aimed at correcting the immunodeficiency. However, as demonstrated in our case, hypogammaglobulinemia does not always resolve following thymectomy, indicating that thymoma management alone may not be sufficient to correct the underlying immunodeficiency. Immunoglobulin replacement therapy, in combination with antimicrobials, remains critical for patients with recurrent infections [2].

*C. coli* infections are rare in immunocompromised patients and can present as acute gastroenteritis or, less commonly, as bacteremia. The presence of lichen planus lesions, as seen in our patient, may facilitate the entry of pathogens into the bloodstream, particularly following gastrointestinal symptoms such as diarrhea. Cutaneous and mucosal lesions because of lichen planus, in immunocompromised patients, can serve as potential entry points for opportunistic infections and, therefore, lead to potentially serious infections [8].

In addition to *C. coli* bacteremia, the patient’s severe COVID-19 infection contributed to her deteriorating condition. COVID-19 has been shown to have a particularly severe impact on patients with compromised immune systems, and the dual burden of viral and bacterial infections can significantly increase mortality risk in these individuals [7,9]. Infection is the principal cause of death in patients with GS, and the prognosis in these populations is generally poorer compared to other adult immunodeficiencies [10].

In the case of our patient, the hypogammaglobulinemia placed her at an increased risk for serious infections, including bacterial and viral pathogens, as it was observed by the concomitant infection by SARS-CoV-2 and *C. coli.* This likely contributed to her increased susceptibility to *C. coli* bacteraemia, when combined with the aggravated SARS-CoV-2 infection, which ultimately led to her worse outcome and death.

## Conclusions

The management of GS requires a multidisciplinary approach, including thymectomy, immunoglobulin replacement, and the early initiation of antiviral and antibacterial therapy, particularly in the presence of recurrent infections. The case presented highlights the importance of considering rare infections such as *C. coli* bacteremia in immunocompromised patients, as well as the increased risk of severe outcomes when combined with viral infections such as SARS-CoV-2. Early identification and tailored therapy can significantly improve the prognosis of these patients.
